# Role of teaching assistants as affective mediators supporting social–emotional project-based service learning

**DOI:** 10.3389/fmed.2025.1685566

**Published:** 2025-12-01

**Authors:** Shih-Chieh Liao, Yueh-Nu Hung, Szu-Han Chen, Yung-Lin Chen, You-Xin Ting, Chia-Rung Chang

**Affiliations:** 1 China Medical University (Taiwan), Taichung, Taiwan; 2 National Taichung University of Education, Taichung, Taiwan

**Keywords:** social and emotional learning, project-based service learning, teaching assistants, affective mediation, SAFE instructional framework, experiential learning, health professions education, student support strategies

## Abstract

**Background:**

Social–emotional learning (SEL) is crucial to the development of professional identity and collaborative competence in medical education. However, research exploring how teaching assistants (TAs) act as affective mediators to support SEL in project-based service-learning (PjBL-SL) is lacking. The present study investigated two research questions: (1) How do the roles and facilitation strategies adopted by TAs in PjBL-SL influence the development of medical students’ SEL competencies? and (2) How do TAs interpret students’ SEL growth trajectories during the course?

**Methods:**

This qualitative study was conducted in a required PjBL-SL course at a Taiwanese medical school. In August 2022, semi-structured interviews were conducted with 11 TAs. Thematic analysis was used to examine their observations and strategies to support student SEL competency development.

**Results:**

The TAs reported observing substantial student growth across all five SEL competencies defined by the Collaborative for Academic, Social, and Emotional Learning: self-awareness, self-management, social awareness, relationship skills, and responsible decision-making. The TAs described how students engaged in perspective-taking, reflective practice, and collaborative problem-solving. The TAs viewed themselves as affective mediators, providing emotional safety, attentive feedback, and tailored guidance throughout the learning process. A dual-layer TA support system—consisting of precourse training grounded in the sequenced, active, focused, explicit principles and ongoing in-course reflective dialogue—was a key enabler of this emotional support role.

**Conclusion:**

TAs in PjBL-SL courses are crucial to supporting medical students’ SEL competency development by offering emotionally attuned, context-sensitive guidance. This study’s dual-layer model of TA preparation and engagement can enhance the emotional dimensions of learning in medical curricula.

## Introduction

Social and Emotional Learning (SEL) is a set of interrelated competencies acquired through observation, imitation, and social participation, enabling students to recognize emotions, manage stress, cultivate empathy, and interact effectively with others (1–3). SEL development begins with self-awareness, advances through emotional regulation and interpersonal skill-building, and ultimately supports responsible decision-making in the face of life and societal challenges (3). The framework proposed by the Collaborative for Academic, Social, and Emotional Learning (CASEL) suggests that SEL develops five core competencies: self-awareness, self-management, social awareness, relationship skills, and responsible decision-making (4–7). Self-awareness involves students’ understanding of their values, interests, and strengths as well as the ability to identify their emotions; self-management refers to the ability to regulate emotions, impulses, and stress while setting and achieving goals; social awareness involves understanding others’ perspectives and differences and demonstrating empathy; relationship skills involve the ability to foster positive interactions and resolve conflicts; and responsible decision-making involves ethical and constructive choices that benefit self and others (8). In medical education, SEL is closely linked to the development of students’ clinical competencies, particularly the competencies of interpersonal communication, emotional regulation, and empathy. These competencies align closely with the six core competencies proposed by the Accreditation Council for Graduate Medical Education and are a foundational component of professional identity formation in the field of medicine (9). Nevertheless, studies in medical education that systematically examine how SEL competencies are cultivated are lacking, particularly empirical studies investigating the influence of instructional strategies on SEL competency development from the perspectives of curriculum design and faculty engagement.

Project-Based Learning (PjBL) is a student-centered instructional model in which learners engage in inquiry centered on a meaningful question or theme. In this approach, instructors offer differentiated guidance and assessment tailored to students’ individual characteristics, fostering exploration and reflection. The learning process culminates in a final presentation that showcases students’ outcomes and growth (10–14). By contrast, Service Learning (SL) is an educational approach that integrates academic goals with community service. SL applies knowledge to real-world social contexts, reflective practice, and the development of professional competence and a sense of social responsibility (15–18). PjBL-SL, which integrates the principles of PjBL and SL, emphasizes active student participation, co-design, and implementation. In this model, instructors and students collaborate in planning projects that respond to community needs. PjBL-SL courses incorporate critical reflection, peer learning, formative assessment, and public dissemination of results. In such courses, social engagement is a vehicle for strengthening learning outcomes and cultivating students’ sense of civic responsibility (11, 18, 19).

In PjBL courses, learning facilitators—instructors and teaching assistants (TAs) —support student development by guiding discussions, assisting with group work, offering timely feedback, and providing emotional support. These practices enhance students’ self-directed learning and problem-solving skills (20–22). As intermediaries between students and the course objectives, TAs interact with learners and may exert a substantial influence on their emotional awareness, interpersonal relationships, and motivation throughout the learning process (21).

In medical education, the combination of PjBL and SL enhances students’ interpersonal communication skills, professional identity, and reflective learning through projects grounded in community needs (18). Despite these advantages, the implementation of combined PjBL-SL courses presents several challenges, such as limited student experience with community engagement, the tendency of learners to lose focus on learning objectives during project execution, and the inability of TAs to provide adequate support without appropriate training. To address these challenges, the present study designed a PjBL-SL course with a dual-layer TA support system grounded in the SAFE (Sequenced, Active, Focused, Explicit) instructional framework. This design reinforced the supportive role of TAs in PjBL-SL and enhanced student SEL outcomes.

This study was conducted within a first-year PjBL-SL course offered by the College of Medicine at China Medical University in Taichung, Taiwan. Through semi-structured interviews with TAs, this study investigated how their roles and facilitation strategies influenced the development of students’ SEL competencies. This study addressed the following research questions:

RQ1: How do the roles and strategies adopted by TAs in PjBL-SL courses influence the development of medical students’ SEL competencies?

RQ2: How do TAs interpret the developmental trajectories of students’ SEL competencies?

The present study contributes to the theoretical understanding of SEL competency cultivation within medical education. Through its model of concrete pedagogical practice grounded in qualitative research, this study demonstrated the critical role of TAs in supporting students’ development of core SEL competencies, specifically emotional awareness, interpersonal communication, empathy, and teamwork. The findings offer insights to inform the design of TA training programs and models of PjBL-SL education to increase the effectiveness of TAs in facilitating the development of student SEL competencies.

## Methods

### Research context and course design

This study focused on the experiences of the TAs in a PjBL-SL course offered in the spring semester of 2022 for first-year medical students at China Medical University. The course included 128 students divided into 11 groups (11 to 13 students per group). The students’ SL projects focused on promoting the health of older adults within the local community of Taichung City where the university is located. Each group was assigned one second-year medical student to serve as a TA. All TAs had completed a SL course during their first year and voluntarily participated. Each TA was involved throughout the entire course process, from project planning, implementation, and reflection to the final presentation, providing continual support and guidance.

### TA training program

To enhance students’ SEL outcomes in the PjBL-SL course, a dual-layer TA support system was applied. This system was designed to systematically train the TAs to function as learning facilitators who provided the students with continued stable support.

To strengthen students’ SEL development, the course incorporated a dual-layer TA support system based on the SAFE instructional framework proposed by CASEL. The SAFE principles provided the theoretical foundation for course design and TA training.

Sequenced learning ensured that course activities followed a structured progression, moving from awareness and planning to real-world engagement, which allowed students to gradually develop communication, empathy, and teamwork skills.

Active learning encouraged student participation in project co-design, community collaboration, and reflective dialogue, fostering ownership and intrinsic motivation.

Focused instruction emphasized specific SEL competencies through targeted feedback and reflective activities.

Explicit guidance clarified the TAs’ facilitative roles and responsibilities, supporting consistent emotional and pedagogical scaffolding throughout the course.

The dual-layer TA support system consisted of two interconnected components: a pre-course workshop and in-course reflection meetings. The workshop equipped TAs with facilitation and emotional support skills grounded in SAFE principles, while the online reflection meetings (conducted via a dedicated LINE group) enabled continuous sharing, feedback, and strategy adjustment during the course.

This integrated design connected the theoretical principles of SAFE with the practical facilitation strategies used by TAs, aligning the instructional design directly with the study’s research questions on how TA roles and strategies influenced students’ SEL development.

### Interview protocol development and credibility assurance

The interview protocol was developed by the research team in June 2022 on the basis of the literature, increasing its content relevance and clarity (15, 16, 18, 20). The interview topics covered three domains: (1) the TAs’ experiences interacting with the students during classroom and SL activities; (2) the support the TAs provided the students in designing and implementing course activities and service projects; and (3) the developmental changes in the students observed by the TAs. To encourage deep reflection, the interview process enabled the participants to ask reverse questions after responding to those posed by the interviewer, fostering two-way communication.

The interviewer was a medical student and coauthor who had completed a SL course. The decision to assign a student rather than a faculty member as the interviewer was made to minimize social desirability bias and enhance the credibility and authenticity of the participants’ responses. Before data collection, the first author, an experienced qualitative researcher, provided intensive training on ethical interviewing, reflexive listening, and probing techniques to ensure data richness and trustworthiness throughout the interviews. In August 2022, the first author trained the interviewer, who conducted pilot interviews with two medical students to examine the credibility, clarity, and data richness of the interview instrument, ensuring that the questions effectively elicited participants’ in-depth reflections and were appropriate for the study context.

### Participants and recruitment

The sample comprised 11 s-year medical students who served as TAs in the 2022 SL course at China Medical University. Data collection continued until no new themes emerged. After the tenth interview, data saturation was reached; one additional interview was conducted to confirm this point, resulting in a total of 11 interviews. Recruitment information was disseminated through a TA-specific LINE group. The participants all voluntarily agreed to be interviewed.

### Ethical considerations and interview procedure

From August to November 2022, the interviewer conducted one-on-one semi-structured interviews with each TA participant in a quiet and private meeting room on the campus of China Medical University. Each interview lasted between 50 and 70 min. Before beginning the interview, the interviewer explained the purpose of the study, the participants’ rights, and the principles of data confidentiality. The participants were assured that participation would not affect their grades or performance evaluations and that they could withdraw at any time. The participants all signed a written informed consent form and agreed to audio recording. Transcripts were produced verbatim and subsequently reviewed by the participants to ensure accuracy before proceeding with data analysis. This study received ethical approval from the Research Ethics Committee of China Medical University (IRB: CRREC-112-035). All participants provided written informed consent, and all procedures involving human participants were conducted in accordance with institutional guidelines and the ethical principles of the Declaration of Helsinki.

### Data analysis and trustworthiness

This study employed reflexive thematic analysis following the six-phase framework proposed by Braun and Clarke (23). The analysis combined inductive and deductive reasoning to identify and interpret patterns of meaning within the interview data. Inductive coding was used to capture participants’ lived experiences, whereas deductive analysis was guided by theoretical perspectives on project-based and service learning to ensure coherence with the research questions.

The original Chinese interview transcripts were analyzed using NVivo 12 software to assist with data management, coding organization, and visualization of thematic relationships. Following Braun and Clarke’s six analytical phases (familiarization, initial coding, theme generation, theme review, definition, and reporting), four researchers independently conducted initial coding and subsequently engaged in iterative and reflexive discussions to review, merge, and refine codes into coherent themes. Through this interpretive and collaborative process, the team developed a comprehensive thematic structure that represented the TAs’ roles, facilitation strategies, and observations of students’ SEL development. For manuscript preparation, relevant excerpts were translated into English by two of the authors, both of whom hold doctoral degrees from U. S. institutions. All quotations were anonymized using coded identifiers (e.g., S#) to protect participant privacy and facilitate analysis.

To ensure trustworthiness, the study followed the qualitative quality criteria proposed by Lincoln and Guba (24), namely credibility, dependability, confirmability, and transferability, rather than quantitative notions of validity or reliability. The research team maintained analytic memos and reflexive notes to document decision-making processes and enhance transparency.

The study also adopted investigator and theoretical triangulation in a reflexive and interpretive manner rather than as a validation procedure. Investigator triangulation was achieved through collaboration among researchers with diverse disciplinary backgrounds, including two educational psychology experts and two medical students, whose dialogues broadened interpretive perspectives and minimized individual bias. Theoretical triangulation was realized by interpreting the data through complementary frameworks of SEL, PjBL, and SL, enriching the analysis and ensuring epistemological coherence.

Triangulation in this study thus served not to “verify” findings statistically but to deepen interpretive understanding through multiple perspectives. Consensus on themes emerged through collaborative coding and interpretive discussion, aligning with the philosophical underpinnings of reflexive thematic analysis.

### Researcher reflexivity

The research team recognized that their disciplinary backgrounds and prior experiences in PjBL-SL could influence data interpretation. The first and corresponding authors are faculty members with extensive experience in medical education and service-learning curriculum design, whereas the other researchers are medical students who previously participated in PjBL-SL courses. This combination of educator and student perspectives provided both insider and outsider viewpoints, enriching the analysis while reducing potential individual bias. Throughout the research process, the team maintained reflexive journals and analytic memos to continually examine their assumptions, positionalities, and potential influence on theme development. Regular team meetings facilitated reflexive dialogue and ensured that interpretations were grounded in participants’ experiences and narratives rather than researchers’ preconceptions.

## Results

The results revealed three major themes and corresponding subthemes (Table 1).

**Table 1:** Themes and subthemes from student responses.

Subtheme	Sample comments (student identifier)	No. of references^a^
Theme 1. TA–Student Relationships		
Peer-like Role Based on Equality and Respect	“The relationship between me and the younger students was like that of friends. I could offer advice and consider things from their perspective.” (S2)	6 (6)
	“I did not want us (TAs) to interact with them (the students) from a position of superiority.” (S7)	
	“The difference between TAs and faculty is that faculty offer professional advice, whereas TAs share nonacademic experiences with students.” (S5)	
Experience-Sharing Mentor Role	“I shared my SL experiences and offered assistance and guidance, which differed from a typical teacher’s role. I aimed to be close to the students and provide practical help.” (S7)	10 (7)
	“The relationship between the TAs and the younger students was one of mutual growth. Because we had experience, we could offer help, but some of the younger students had different perspectives and social experiences that gave the TAs opportunities to reflect and learn as well.” (S10)	
	“To be honest, we are only 1 year apart in age, and some of the younger students actually had more life experience [than we did]. We also learned a lot from them. So, I think the term ‘friends’ best describes the relationship between the students and TAs.” (S8)	
Theme 2. TA Facilitation Strategies		
Guiding Rather Than Instructing	“My primary teaching or facilitating method was to intervene only when students encounter problems; doing so enabled them to discuss and develop their own solutions. I did not impose restrictions or dictate what could not be done; instead, I gave them the space to generate their own ideas.” (S4)	19 (9)
	“Reminding students, encouraging active participation, and interacting with them—these strategies support their learning.” (S1)	
	“I would not directly tell them which direction to take, because I believe that critical thinking is essential in SL. So, I mostly waited for them to raise questions and offered suggestions or ideas.” (S10)	
	“I mostly took on a passive role, letting the students take the lead. If they encountered problems, I would share my perspective, which they could choose whether to adopt.” (S8)	
Emotional Support and Encouragement	“What impressed me most about being a TA was being able to accompany the students throughout the course, gaining a deep understanding of their situations and offering more effective support.” (S7)	4 (4)
	“I regularly checked on student progress and group dynamics, reminded the students of urgent tasks, and helped them create schedules—monitoring their progress and reviewing their outcomes.” (S6)	
	“The TAs joined the student group chats (on the LINE platform), which enabled us to track their progress. This enabled us to provide timely responses when problems arose. I also kept in touch with the group leaders to monitor their status.” (S9)	
	“When the students encountered problems, I would remind them to first consider how to resolve the problems rather than avoid them.” (S3)	
Theme 3. Student Learning Outcomes		
Reflection and Perspective-Taking	“When designing service activities, the students realized that they had previously approached problems only from their own perspectives; it was only when they interacted directly with the service recipients that they came to understand their needs and challenges.” (S3)	23 (10)
	“The medical students developed empathy, practiced active listening, and made timely adjustments to bridge communication gaps.” (S2)	
	“When faced with the gap between expectations and reality, I initially felt confused and powerless, but this helped me learn how to adapt and set clearer, more appropriate goals and action plans on the basis of the recipients’ needs.” (S6)	
	“Through the service activities, the younger students gained a deep understanding of the course’s goals and learned to see things from different perspectives.” (S11)	
	“The most crucial part of an SL course is the hands-on experience. Let the students engage in the activity first, then gradually discover its value—learning through mistakes, learning through action.” (S11)	
Civic Engagement	“Through the SL process, the students gained hands-on learning experience and developed a sense of civic engagement and the ability to solve problems.” (S6)	8 (6)
	“This SL experience helped the students understand the concept of mutual benefit—when the service recipients received assistance, the students gained invaluable experience.” (S10)	
	“SL courses prompt students to reflect on their lives and feel gratitude and encourage them to help older individuals when they are capable.” (S2)	
Social Problems	“SL enabled the students to identify societal problems, independently develop solutions, and implement them.” (S9)	5 (4)
	“During our discussions and actions, the students and I gradually became aware of the root causes of these problems [we sought to address], and began to consider how real-world actions could drive change.” (S8)	
	“After participating in the SL project, the students realized that some problems could not be resolved through individual efforts alone; rather, they require the involvement of greater resources and collective societal efforts.” (S9)	
	“The students were encouraged to independently reflect on social problems or societal needs, discuss with group members how they could address them, and implement actions to resolve these challenges.” (S8)	
Teamwork	“The students demonstrated practical skills, critical thinking, and the ability to collaborate during project design and implementation.” (S8)	8 (6)
	“This experience helped me realize that teamwork is not just about dividing tasks—it is about mutual support and coordination, and everyone’s contribution matters.” (S3)	
	“Through hands-on service, the students gained practical experience, developed problem-solving abilities, increased their sense of civic engagement and teamwork, and deepened their understanding of social problems.” (S6)	
	“Teamwork is a key soft skill that students develop through SL training.” (S8)	
	“Everyone gathered to rehearse the performance parts of the service activities or to discuss the lesson plan content.” (S10)	
Planning	“The process of writing the proposal helped the students understand that SL is not driven by enthusiasm alone; it requires thorough planning and execution.” (S2)	4 (2)
	“The students often initially developed proposals that were overly idealistic… but they learned to revise them to make the plans more achievable—and that, too, was a valuable learning experience.” (S5)	
	“I believe the younger students grew the most in their ability to write project proposals. They learned how to plan something and concretely articulate it in a written proposal.” (S4)	
Responsibility and Sense of Achievement	“The course cultivated the students’ ability to collaborate and their sense of responsibility, enabling them to design service plans that met the needs of the target population, learning through hands-on engagement.” (S3)	4 (3)
	“When we saw that the service recipients genuinely benefited from the students’ efforts, the students felt a deep sense of accomplishment. That [sense of accomplishment] gave them greater motivation to invest in future learning and professional endeavors.” (S5)	
	“The younger students gained a sense of achievement and motivation during the SL process, in addition to practical experience and skills.” (S11)	

a The first number indicates the total number of times the theme or subtheme was mentioned; the number in parentheses represents the number of students who mentioned the theme or subtheme (students may mention the same theme or subtheme multiple times).

### Theme 1: TA-student relationships (two subthemes)

#### Peer-like role based on equality and respect

The TAs developed a peer-like relationship with the students that was grounded in mutual respect and equality. Unlike conventional teacher-student dynamics, the relationship between the TAs and the students emphasized egalitarian interactions and reciprocal respect. As one TA noted, *The relationship between me and the younger students was like that of friends. I could offer advice and consider things from their perspective.* (S2) Another TA echoed this view, emphasizing the importance of maintaining equality rather than hierarchy in TA–student interactions: *I did not want us (TAs) to interact with them (the students) from a position of superiority.* (S7) Together, these accounts highlight that the peer-like relationship between TAs and students was characterized by empathy, equality, and mutual learning, distinguishing it from traditional teacher-centered relationships.

#### Experience-sharing mentor role

The TAs supported the students by sharing their own experiences with SL. Drawing on these experiences, they provided concrete advice and guidance to help the students navigate challenges and uncertainties. One TA explained, *I shared my SL experiences and offered assistance and guidance, which differed from a typical teacher’s role. I aimed to be close to the students and provide practical help.* (S7) Another TA further emphasized that this mentoring relationship was mutually beneficial, describing how interactions with the younger students also promoted the TAs’ own reflection and learning: *The relationship between the TAs and the younger students was one of mutual growth. Because we had experience, we could offer help, but some of the younger students had different perspectives and social experiences that gave the TAs opportunities to reflect and learn as well.* (S10) Together, these accounts demonstrate that the TAs acted not only as mentors who shared experiences and practical advice but also as co-learners who engaged in reciprocal growth with the students.

### Theme 2: TA facilitation strategies (two subthemes)

#### Guiding rather than instructing

The TAs adopted a facilitative approach that prioritized prompting and guidance over direct instruction, encouraging the students to think independently and solve problems autonomously. One TA observed, *My primary teaching or facilitating method was to intervene only when students encounter problems; doing so enabled them to discuss and develop their own solutions. I did not impose restrictions or dictate what could not be done; instead, I gave them the space to generate their own ideas.* (S4) Rather than offering immediate answers, the TAs stepped back and intervened only when necessary. This hands-off approach enabled the students to engage in problem-solving through discussion and creative thinking. As another TA reflected, *Reminding students, encouraging active participation, and interacting with them—these strategies support their learning.* (S1)

#### Emotional support and encouragement

The TAs supported the students emotionally by offering companionship and feedback that increased motivation and provided reassurance. Their presence fostered reciprocal emotional bonds. One TA shared, *What impressed me most about being a TA was being able to accompany the students throughout the course, gaining a deep understanding of their situations and offering more effective support.* (S7) In addition, another TA emphasized proactive engagement and consistent guidance to sustain students’ motivation: *I regularly checked on student progress and group dynamics, reminded the students of urgent tasks, and helped them create schedules—monitoring their progress and reviewing their outcomes.* (S6) Together, these reflections illustrate that emotional support in the PjBL–SL context extended beyond empathy and care to include active monitoring, encouragement, and timely feedback that strengthened students’ confidence and perseverance.

### Theme 3: student learning outcomes (six subthemes)

#### Reflection and perspective-taking: students develop a multidimensional understanding of others’ perspectives

One TA observed, *When designing service activities, the students realized that they had previously approached problems only from their own perspectives; it was only when they interacted directly with the service recipients that they came to understand their needs and challenges*. (S3) This shift from egocentric thinking enabled the students to grow through the challenges and failures they encountered during the service experience, prompting deep reflection on their personal values and professional identities. One student, after facing setbacks in the course of service implementation, shared the following insights with their group TA: *When faced with the gap between expectations and reality, I initially felt confused and powerless, but this helped me learn how to adapt and set clearer, more appropriate goals and action plans on the basis of the recipients’ needs*. (S6) The TAs also affirmed that the process of reflection and perspective-taking during service activities considerably enhanced the students’ social awareness. This process exemplified learning by doing. As one TA noted, The most crucial part of an SL course is the hands-on experience. Let the students engage in the activity first, then gradually discover its value—learning through mistakes, learning through action. (S11)

#### Civic engagement: experiencing public responsibility through action

Service activities were not merely components of the course but also served as opportunities for the students to engage with societal problems. As one TA remarked, *Through the SL process, the students gained hands-on learning experience and developed a sense of civic engagement and the ability to solve problems.* (S6) By actively participating in service projects, the students strengthened their awareness of their social responsibility and deepened their concern for social problems. Throughout the learning process, they experienced the value of service, learned how to interact with society, and began to explore their roles in contributing to social change. One TA noted, *This SL experience helped the students understand the concept of mutual benefit—when the service recipients received assistance, the students gained invaluable experience.* (S10)

#### Social problems: from awareness to action-oriented thinking

Through SL, the students deepened their awareness of social problems and enhanced their problem-solving skills. One TA remarked, *SL enabled the students to identify societal problems, independently develop solutions, and implement them.* (S9) As the students engaged in service activities, they gradually developed an awareness of structural inequalities and social disparities, prompting reflection on their individual responsibilities and potential to contribute to social change. As one TA shared, *During our discussions and actions, the students and I gradually became aware of the root causes of these problems [we sought to address], and began to consider how real-world actions could drive change.* (S8) Another TA added, *After participating in the SL project, the students realized that some problems could not be resolved through individual efforts alone; rather, they require the involvement of greater resources and collective societal efforts.* (S9)

#### Teamwork: learning to collaborate, delegate, and support

One TA observed, *The students demonstrated practical skills, critical thinking, and the ability to collaborate during project design and implementation*. (S8) During collaboration, the students learned how to work effectively with others to accomplish shared goals. They developed key interpersonal skills such as communication and coordination. Through teamwork, they learned to integrate diverse viewpoints, distribute tasks, and cooperatively pursue the objectives of their service projects. As one student remarked to a TA, *This experience helped me realize that teamwork is not just about dividing tasks—it is about mutual support and coordination, and everyone’s contribution matters*. (S3) Hence, participating in the design and implementation of service projects enhanced the students’ communication skills, problem-solving abilities, and group cohesion. Another TA noted, Through hands-on service, the students gained practical experience, developed problem-solving abilities, increased their sense of civic engagement and teamwork, and deepened their understanding of social problems. (S6)

#### Practical planning: drafting and adapting service project proposals

In the PjBL-SL course, the students learned to design and draft concrete and feasible SL project proposals—an outcome indicative of their self-management abilities. One TA observed, *The process of writing the proposal helped the students understand that SL is not driven by enthusiasm alone; it requires thorough planning and execution.* (S2) The students also learned how to adapt their plans to real-world constraints to enhance feasibility and relevance. As another TA noted, *The students often initially developed proposals that were overly idealistic… but they learned to revise them to make the plans more achievable—and that, too, was a valuable learning experience.* (S5).

#### Responsibility and sense of achievement: increasing confidence and motivation through accountability

Throughout the SL process, the students adjusted their service activities to ensure smoother implementation—demonstrating their sense of responsibility. As one TA explained, *The course cultivated the students’ ability to collaborate and their sense of responsibility, enabling them to design service plans that met the needs of the target population, learning through hands-on engagement.* (S3) Upon completing their service projects, the students experienced a strong sense of achievement and pride, which enhanced their self-confidence and motivation to learn. One TA shared, *When we saw that the service recipients genuinely benefited from the students’ efforts, the students felt a deep sense of accomplishment. That [sense of accomplishment] gave them greater motivation to invest in future learning and professional endeavors.* (S5) In addition to feeling satisfied with their academic outcomes, the students learned valuable lessons regarding the effects of their actions on others and cultivated a strong sense of social responsibility.

To synthesize the findings regarding the three major themes and six dimensions of student SEL outcomes, the research team developed a conceptual process model illustrating how the TAs facilitated the students’ SEL growth trajectories in the course through targeted strategies. The model and its implications are discussed in the following section.

## Discussion

### PjBL-SL enhances core SEL competencies: observations and interpretations of TAs

The TAs observed clear growth in students’ core SEL competencies throughout the PjBL-SL course. Through field-based service and reflective practice, students developed greater social awareness and a deeper understanding of their societal roles and responsibilities, fostering social and professional identity. Direct, authentic interactions with service recipients helped students move beyond abstract knowledge to real-world engagement. Action-based learning nurtured empathy, a care-oriented mindset, and respect for cultural diversity. Teamwork further enhanced communication, coordination, and problem-solving skills. Planning and implementing service activities provided authentic opportunities to practice self-management and responsible decision-making.

Beyond describing student outcomes, these findings highlight that SEL development within PjBL-SL was not incidental but systematically scaffolded through sequenced, active, focused, and explicit (SAFE) learning opportunities. Students progressed from emotional awareness to social engagement through iterative cycles of experience, reflection, and re-engagement, demonstrating the integrative operation of SAFE and SEL principles in authentic service contexts.

Overall, the TAs found that students’ SEL growth aligned with CASEL’s five core competencies: self-awareness, self-management, social awareness, relationship skills, and responsible decision-making, which were acquired through experiential learning and meaningful social interaction.

### Motivational process of SEL competency development in PjBL-SL: the TA as an innovative affective mediator

In addition to supporting the design and implementation of service-learning projects, TAs served as affective mediators—crucial for fostering students’ SEL development. With high emotional sensitivity and responsiveness, they detected students’ emotional shifts and needs, offering timely support that enhanced emotional regulation, communication, and empathy (Theme 1). Through a supportive approach, TAs facilitated student reflection, collaboration, and engagement with real-world contexts, promoting habits of responsible decision-making (Theme 2).

They created emotionally safe and trusting environments where students could express emotions, confront setbacks, and remain engaged. When difficulties arose, TAs provided reassurance, helping students reframe experiences, adjust strategies, and sustain intrinsic motivation and self-efficacy.

Importantly, the how and why of TAs’ mediating function can be understood through both psychological and cultural lenses. Psychologically, TAs enacted emotional scaffolding by balancing challenge and support to sustain students’ motivation and emotional balance. Culturally, within the Taiwanese educational context that values hierarchical respect and collective harmony, TAs bridged the relational distance between teachers and students, humanizing learning interactions and allowing emotions to surface safely. Their mediating behaviors thus reflected contextually grounded emotional labor, transforming hierarchical structures into supportive communities of shared growth.

Beyond logistical and instructional roles, TAs actively contributed to students’ emotional and cognitive development. Through sustained interaction, they observed growth in students’ emotional regulation, coordination, and responsibility, offering context-sensitive, developmentally attuned guidance to support transformative learning.

### Strengthening the affective mediating role of TAs: the dual-layer TA support system

The dual-layer TA support system enabled the TAs to effectively fulfill their roles as affective mediators. This system comprised two components: a precourse TA workshop and in-course online reflection meetings. The precourse workshop was grounded in the SAFE principles proposed by CASEL (25) and provided the TAs with a structured and theoretically informed foundation in instructional and facilitation strategies. The in-course reflection meetings provided a mechanism for peer-to-peer experience sharing and emotional support that enhanced the TAs’ professional reflection and pedagogical adjustment abilities.

**Figure 1:**
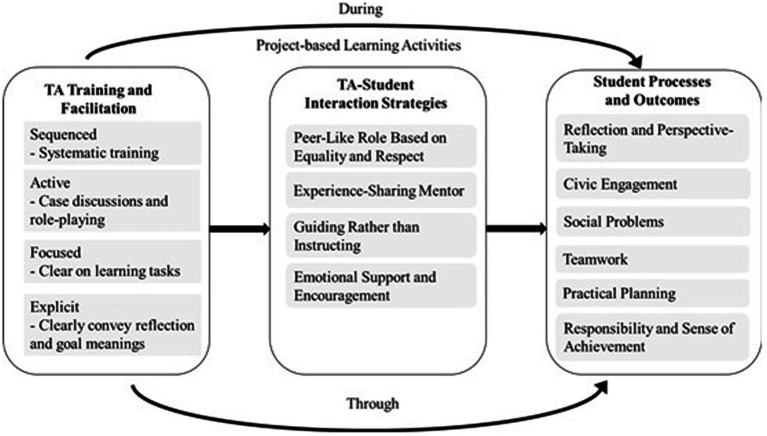
From teaching assistant training to student social and emotional learning outcomes: a conceptual model of facilitated project-based service-learning.

On the basis of the findings, this study developed a theoretical model to illustrate how the TA support system integrated precourse structured training with in-course facilitation to support the development of students’ SEL competencies (Figure 1). The component labeled “during Project-Based Learning Activities” refers to the facilitation strategies the TAs acquired during training, such as discussion facilitation, teamwork coordination, and timely feedback and emotional support provision, which they actively applied throughout the students’ learning processes. These strategies enabled the TAs to engage in affective mediation to meet the students’ needs. The emphasis in the “during Project-Based Learning Activities” component lay in the ongoing and responsive interactions between the TAs and students, through which the students engaged emotionally and socially in participatory learning environments. By contrast, the component “through Project-Based Learning Activities” underscored the continued pervasive role of TA facilitation strategies throughout the PjBL process. Through consistent in-context intervention and support, the TAs guided the students to acquire SEL competencies through iterative cycles of action and reflection.

Theoretical integration of these findings suggests that the dual-layer support system not only enhances TAs’ pedagogical and affective competencies but also reinforces a cyclical model of learning mediation. Through structured preparation and ongoing reflection, TAs continuously internalized and applied SEL-oriented facilitation strategies that shaped their own social–emotional growth as well as that of their students.

Thus, the TA support system represents more than a procedural design; it embodies a SAFE-aligned mechanism for cultivating emotionally intelligent educators capable of mediating learning through empathy, responsiveness, and reflection. Within the broader context of medical education, this model illustrates how structured, relationally grounded support can bridge cognitive learning and affective growth, contributing to the formation of compassionate, self-aware, and socially responsible professionals.

### Responses to research questions

#### Research question 1

Throughout the PjBL-SL course, TAs fulfilled multiple roles—as peers, experience-sharing mentors, and facilitators—to holistically support students’ SEL development. As peers, they created an environment of equality and respect that encouraged emotional expression and enhanced empathy. As mentors, they shared SL experiences to foster students’ self-management and responsibility. As facilitators, they guided project-based teamwork to cultivate responsible decision-making and civic engagement.

TAs also acted as affective mediators, using emotional sensitivity and timely responses to build a safe, trusting space for learning. This emotional scaffolding promoted students’ emotional regulation, self-efficacy, and reflective capacity, enabling them to internalize SEL competencies and apply them in real-world contexts. The effectiveness of this support was linked to the TAs’ structured pre-course training based on CASEL’s SAFE principles (25), which strengthened their facilitation skills.

#### Research question 2

TAs observed that students gradually internalized SEL core competencies through experiential learning. Authentic interactions, teamwork, and perspective-taking enabled students to acquire and demonstrate the five CASEL-defined competencies. This growth was evident in behavioral changes and the emergence of social responsibility and professional identity. Students moved from abstract knowledge to real-world application, deepening empathy, ethical awareness, and cultural respect. These experiences also fostered advanced collaboration, communication, and conflict resolution skills.

### Triangulation and dialogue with the literature: alignment and innovation

This study adopted a qualitative interview-based analysis, whereas Liao et al. (18) employed a quantitative survey method. Despite these methodological differences, both studies agree in their findings: In PjBL-SL courses, students sharpen their communication skills, collaborative abilities, and sense of civic engagement through cooperation, engagement, and reflection. The findings of the present study are consistent with those of the literature (26–31), which suggests that SL enables students to apply SEL competencies in the real world through fostering connections with the community. These connections can be transformed into long-term learning motivations and a commitment to social engagement. Additionally, the results of this study align with those of other studies (32–34) indicating that learning environments characterized by care and respect enhance students’ sense of safety and participation. Such environments strengthen intrinsic motivation and a sense of responsibility, enhancing overall learning outcomes.

The primary innovation of this study lies in its positioning of TAs as the central observers and interpreters of student experiences. Through close, sustained interactions, the TAs witnessed the development of the students’ SEL competencies. This study verified the effectiveness of PjBL-SL in promoting SEL and mapped a developmental process in which the TAs initiated and guided student learning through facilitation strategies, which were internalized by the students through active participation, deep reflection, and reciprocal feedback. This process model, centered on the role of TAs as affective mediators, offers a valuable theoretical reference for the design of SL curricula and TA training systems. The model can be applied to interdisciplinary and multilevel educational contexts to extend the applications of PjBL-SL in higher education.

### Limitations and recommendations for further research

This study has several limitations inherent to qualitative inquiry. First, it was conducted with a small group of teaching assistants from a single course at one institution; therefore, the findings reflect context-specific perspectives and may have limited transferability to other disciplines or course designs. Second, although reflexive discussions and collaborative interpretation were used to enhance credibility and trustworthiness, the researchers’ prior involvement in the SL course may have influenced data interpretation. Finally, this study did not include follow-up interviews to explore the long-term influence of PjBL–SL on students’ or TAs’ professional growth. Future research could adopt a longitudinal and multi-site design to further explore how project-based service learning shapes participants’ clinical practice, self-identity, and professional competence across different educational settings.

## Conclusion

When course design effectively integrates the theoretical foundations of PjBL and SL, courses can provide a fertile environment for students to develop SEL competencies. However, the presence of TAs equipped with SAFE facilitation skills is critical to enhancing the development of SEL competencies. TAs must assume multiple roles in PjBL-SL courses—as facilitators, mentors, and emotional supporters—functioning as affective mediators throughout the learning process. This role is vital to cultivating a learning environment characterized by safety, respect, and engagement that strengthens students’ reflections, emotional growth, and sense of social responsibility.

The TA facilitation process model proposed in this study (Figure 1) addresses a gap in the literature on SEL and SL, which rarely comprehensively explores the role of TAs. The model proposed in the present study offers an empirically grounded instructional framework to inform TA training and curriculum development. The model also provides educators with a systematic approach to cultivating TAs’ facilitation competence and emotional support skills, enhancing the effectiveness of SEL-oriented courses. The findings of this study can be applied to other areas in medical and health education (e.g., dentistry or nursing) and can serve as the foundation for developing TA behavior observation indicators, evaluation instruments, and cross-cultural longitudinal studies on SEL. The results of this study advance the practical implementation and theoretical development of integrated PjBL-SL and SEL pedagogies.

## Data Availability

The raw data supporting the conclusions of this article will be made available by the authors, without undue reservation.

## References

[1] Wigelsworth M . Making a case for core components: new frontiers in SEL theory, research, and practice. Sch Psychol Rev. (2024) 53:593–606. doi: 10.1080/2372966X.2021.2004863

[2] Dusenbury L . An examination of frameworks for social and emotional learning (SEL) reflected in state K–12 learning standards. Measuring SEL Using Data Inspire Pract. (2019) 3:1–29.

[3] Frye KE Boss DL Anthony CJ Du H Xing W. Content analysis of the CASEL framework using K–12 state SEL standards. Sch Psychol Rev. (2024) 53:208–22. doi: 10.1080/2372966X.2022.2030193

[4] Elias MJ Leverett L Duffell JC Humphrey N Stepany C Ferrito J. Integrated SEL with related prevention and youth development approaches In: Durlak JA Domitrovich CE Weissberg RP Gullotta TP, editors. Handbook of social and emotional learning. New York: Guilford Press (2015). 33–49.

[5] CASEL. Effective social and emotional learning programs: pre-school and elementary school education. Chicago: CASEL (2013).

[6] CASEL. Effective social and emotional learning programs: middle and high school education. Chicago: CASEL (2015).

[7] Weissberg RP Durlak JA Domitrovich CE Gullotta TP. Social and emotional learning: past, present, and future In: Durlak JA Domitrovich CE Weissberg RP Gullotta TP, editors. Handbook of social and emotional learning: research and practice. New York: Guilford Press (2015). 3–19.

[8] Mahoney JL Weissberg RP Greenberg MT Dusenbury L Jagers RJ Niemi K . Systemic social and emotional learning: promoting educational success for all preschool to high school students. Am Psychol. (2021) 76:1128–42. doi: 10.1037/amp0000701 33030926

[9] Hsu WC Fuh LJ Liao SC. Tickling the heart: integrating social emotional learning into medical education to cultivate empathetic, resilient, and holistically developed physicians. Front Med. (2024) 11:1368858. doi: 10.3389/fmed.2024.1368858 PMC10944992 38500950

[10] Barak M. Problem-, project- and design-based learning: their relationship to teaching science, technology and engineering in school. J Probl Based Learn. (2020) 7:94–7. doi: 10.24313/jpbl.2020.00227

[11] Baran E Correia AP. Student-led facilitation strategies in online discussions. Distance Educ. (2009) 30:339–61. doi: 10.1080/01587910903236510

[12] Knoll M. The project method project learning method: its vocational education origin and international development. J Ind Teach Educ. (1997) 34:59–80.

[13] Mergendoller JR Larmer J. Why we changed our model of the “8 essential elements of PBL”. Available from: https://my.pblworks.org/resource/blog/why_we_changed_our_model_of_the_8_essential_elements_of_pbl.

[14] Pucher R Lehner M. Project-based learning in computer science: a review of more than 500 projects. Procedia Soc Behav Sci. (2011) 29:1561–6. doi: 10.1016/j.sbspro.2011.11.398

[15] Liao SC Hsu LC Lung CH. Early patient contact course: “be a friend to patients”. Med Educ. (2008) 42:1136–7. doi: 10.1111/j.1365-2923.2008.03202.x 19141021

[16] Gresh A LaFave S Thamilselvan V Batchelder A Mermer J Jacques K . Service learning in public health nursing education: how COVID-19 accelerated community-academic partnership. Public Health Nurs. (2021) 38:248–57. doi: 10.1111/phn.12796 32876353

[17] Yang YS Liu PC Lin YK Lin CD Chen DY Lin BYJ. Medical students’ preclinical service-learning experience and its effects on empathy in clinical training. BMC Med Educ. (2021) 21:301. doi: 10.1186/s12909-021-02739-z 34039327 PMC8157642

[18] Liao SC Lee MR Chen YL Chen HS. Application of project-based service-learning courses in medical education: trials of curriculum designs during the pandemic. BMC Med Educ. (2023) 23:696. doi: 10.1186/s12909-023-04671-w 37740242 PMC10517514

[19] Murray J. Student-led action for sustainability in higher education: a literature review. Int J Sustain High Educ. (2018) 19:1095–110. doi: 10.1108/IJSHE-09-2017-0164

[20] Miller A. Tips for combining project-based and service learning. Available from: https://www.edutopia.org/article/tips-combining-project-based-and-service-learning-andrew-miller.

[21] Kokotsaki D Menzies V Wiggins A. Project-based learning: a review of the literature. Improv Sch. (2016) 19:267–77. doi: 10.1177/1365480216659733

[22] Herrera X Nissen J Van Dusen B. Student outcomes across collaborative learning environments. arXiv. (2018):1808.07076.

[23] Braun V Clarke V. Thematic analysis In: Cooper H Camic PM Long DL Panter AT Rindskopf D Sher KJ, editors. APA handbook of research methods in psychology, vol. 2. Washington, DC: APA (2012). 57–71.

[24] Lincoln YS Guba EG. Naturalistic inquiry. Beverly Hills (CA): Sage Publications (1985).

[25] Durlak JA Weissberg RP Dymnicki AB Taylor RD Schellinger KB. The impact of enhancing students’ social and emotional learning: a meta-analysis of school-based universal interventions. Child Dev. (2011) 82:405–32. doi: 10.1111/j.1467-8624.2010.01564.x 21291449

[26] Gregory A Fergus E. Social and emotional learning and equity in school discipline. Future Child. (2017) 27:117–36. doi: 10.1353/foc.2017.0006

[27] Jagers RJ Rivas-Drake D Williams B. Transformative social and emotional learning (SEL): toward SEL in the service of educational equity and excellence. Educ Psychol. (2019) 54:162–84. doi: 10.1080/00461520.2019.1623032

[28] Celio CI Durlak J Dymnicki A. A meta-analysis of the impact of service-learning on students. J Exp Educ. (2011) 34:164–81. doi: 10.1177/105382591103400205

[29] Elias MJ Zins JE Weissberg RP Frey KS Greenberg MT. Promoting social and emotional learning: guidelines for educators. Alexandria (VA): ASCD (1997).

[30] Delpit L. Other people’s children: cultural conflict in the classroom. New York: Norton (2006).

[31] Jennings T Minnici A Yoder N. Creating the working conditions to enhance teacher social and emotional well-being In: Osher D Mayer M Jagers R Kendziora K Wood L, editors. Keeping students safe and helping them thrive. Santa Barbara: Praeger/ABC-CLIO (2019). 210–39.

[32] Durlak JA Mahoney JL Boyle AE. What we know, and what we need to find out about universal, school-based social and emotional learning programs for children and adolescents: a review of meta-analyses and directions for future research. Psychol Bull. (2022) 148:765–82. doi: 10.1037/bul0000383

[33] Cipriano C Strambler MJ Naples LH Ha C Kirk M Wood M . The state of evidence for social and emotional learning: a contemporary meta-analysis of universal school-based SEL interventions. Child Dev. (2023) 94:1181–204. doi: 10.1111/cdev.13968 37448158

[34] Greenberg MT Brown JL Abenavoli RM. Teacher stress and health: effects on teachers, students, and schools. University Park (PA): Edna Bennett Pierce Prevention Research Center, Pennsylvania State University (2016).

